# Developing and implementing Pediatric Nephrology Milestones 2.0 as an efficient tool for trainee evaluation and just-in-time feedback

**DOI:** 10.1186/s12909-024-06627-0

**Published:** 2025-03-04

**Authors:** Caroline V. Jackson, Rebecca Hjorten, Laura Edgar, Beatrice Goilav, Roshan P. George, Darcy K. Weidemann

**Affiliations:** 1 University of Washington & Seattle Children’s Hospital, Seattle, WA USA; 2 University of Alabama Birmingham & Children’s of Alabama, Birmingham, AL USA; 3 Accreditation Council for Graduate Medical Education, Chicago, Il, USA; 4 Albert Einstein College of Medicine & Children’s Hospital and Montefiore, Bronx, NY USA; 5 Emory School of Medicine & Children’s Healthcare of Atlanta, Atlanta, GA USA; 6 University of Missouri-Kansas City School of Medicine & Children’s Mercy, Kansas City, MO USA

**Keywords:** Milestones 2.0, Pediatric nephrology, Fellowship, Medical education, Evaluation, Feedback, ACGME

## Abstract

In 2013, the Accreditation Council for Graduate Medical Education (ACGME) implemented Milestones 1.0 as a tool to assess trainee progress towards readiness for independent practice. Critiques of Milestones 1.0 suggested its complexity made the tool difficult to quickly understand and implement in a standardized fashion. This was particularly challenging among subspecialties due to inherent differences in clinical practice settings and make-up of procedural and patient care needs. In response, ACGME launched Milestones 2.0 in 2016 to harmonize competencies applicable to all subspecialties and develop new subspecialty specific competencies to facilitate precise feedback on subspecialty specific content domains. We describe how the Pediatric Nephrology Subspecialty Milestones were developed by a working group of pediatric nephrologists, fellows, and members of the ACGME. We highlight how this revision supports a growth-focused educational environment and equitable evaluation process. We describe how some institutions have used Milestones 2.0 to create just-in-time as well as summative feedback tools that quickly translate into individualized learning goals and guidance for programmatic improvements.

## Background

One of the most significant changes in medical training in the past two decades was the development of competency based medical education (CBME). CBME is an outcomes-based approach that utilizes competencies or observable abilities in the assessment of learners [1]. The Accreditation Council for Graduate Medical Education (ACGME) and American Board of Medical Specialties (ABMS) introduced core competencies in 1999 to provide a structured framework for trainee assessment [2, 3] and represent skills that all physicians must master, regardless of specialty. The six competencies are patient care and procedural skills (PC), medical knowledge (MK), systems-based practice (SBP), practice-based learning and improvement (PBLI), professionalism (PROF) and interpersonal communication skills (ICS). The Milestones were subsequently introduced in 2013 to integrate the competencies into subspecialty training and allow structured assessment of a trainee’s progress towards becoming an independent clinician in their field. Milestones are arranged into levels 1 to 5 synonymous with moving from novice to expert fellow in a subspecialty. Levels do not correspond to the learner’s postgraduate year of training. The program’s Clinical Competency Committee selects the milestone levels that best describe each learner’s current performance, abilities, and attributes for each subcompetency. These assessments then serve as a tool to assess progress and detect any signs of deviation from the expected yet flexible progression towards independent practice as learners gain skills [4].

Within several years of Milestones 1.0 implementation, feedback from the medical community demonstrated a need for an iterative improvement cycle. Critiques included concerns that the Milestones were too lengthy and that their complex wording was difficult to interpret and understand [5, 6]. Milestones were felt to be particularly difficult to utilize effectively in the sub-specialty setting. Given the inherent variation in frequency of procedures, patient care settings, and specialized medical knowledge needed in each subspecialty, a one-size-fits-all approach was cumbersome and time-consuming. The complex language of the Milestones hampered the creation of a shared mental model, leading to variations in implementation between programs. Similarly, trainees found it difficult to use Milestones 1.0 feedback to make practical adjustment and create specific goals for improvement [7].

This feedback was the inspiration for the formal initiation of Milestones 2.0 creation in 2016. A major focus of the revision was to find balance between maintaining consistent expectations and benchmarks for all physicians across specialties while permitting unique and individualized learning benchmarks that represent the priorities of particular subspecialties [2, 8].

Milestones 2.0 are intended as a framework to guide delivery of direct, simple, non-biased, and effective feedback that fellows can more easily understand and translate into individualized and actionable learning goals [2, 8]. Review of compiled Milestone 2.0 results can additionally be used for programmatic improvement by identifying gaps in the educational curriculum or clinical exposure within a particular fellowship.

This manuscript describes the creation of Milestones 2.0 for pediatric nephrology and how the Milestones tool can be implemented by training programs to provide both just-in-time and summative feedback for pediatric nephrology fellows.

## Methods

### Developing milestones 2.0

Milestones 2.0 was launched by the ACGME in 2016. One key goal was to harmonize and align the competencies encompass skills that are applicable among all pediatric subspecialties. A second aim was to improve the Milestones’ ability to provide more precise feedback on subspecialty-specific content domain areas [2]. The first step was to develop harmonized milestones for interpersonal communication skills (ICS), practice-based learning and improvement (PBLI), professionalism (PROF), and systems-based practice (SBP) as these competencies were felt to pertain to all physicians regardless of subspecialty. Next, specialty-specific working groups were convened via public call for volunteers. Working groups were tasked with developing subspecialty content for the medical knowledge (MK) and patient care (PC) competencies, as well as creation of a robust Supplemental Guide. The goal of the Supplemental Guide was to foster development of the shared mental model across programs within each specialty and provide specific examples of milestone achievements to aid in implementation of Milestones 2.0.

Members for the pediatric nephrology working group were recruited through solicitations within the Association of Pediatric Program Directors (APPD), American Society of Pediatric Nephrology (ASPN), Council of Pediatric Subspecialties (COPS), and American Board of Pediatrics (ABP). The final group was composed of three pediatric nephrology fellows (two in their third year of training and one in their second) and nine pediatric nephrology faculty. Oversight of the group was managed by three representatives from the ACGME to facilitate discussion and review. The first phase of the revision involved a review and update of the Harmonized Milestones including creation of specific pediatric nephrology examples demonstrating learner progression through these subcompetencies. This work was done via a combination of virtual pre-work and an in-person meeting at ACGME Headquarters in Chicago, IL.

Next, the working group began updating the PC and MK competencies to reflect the specific skill sets needed for a pediatric nephrologist to independently care for patients with a wide range of kidney conditions (Table 1). Throughout this process the overarching goal was that, though the MK and PC subcompetencies would vary between subspecialties, the degree of progression would be standardized such that Level 4 is a graduation goal (but not requirement).

**Table 1:** Pediatric nephrology core competencies and subcompetencies of Milestones 2.0

Core Competency	Subcompetency
**Patient Care (PC)**	
	Organization and Prioritization of Patient Care
	Acute Kidney Injury
	Chronic Dialysis Therapy
	Chronic Kidney Disease
	Transplant
	Fluids, Electrolytes, and Acid–base Disorders
	Hypertension
	Glomerular Disease
	Competence in Procedures
**Medical Knowledge (MK)**	
	Clinical Reasoning
	Physiology and Pathophysiology
**Systems-Based Practice (SBP)**	
	Patient Safety
	Quality Improvement
	System navigation for Patient Centered Care – Coordination of Care
	System navigation for Patient Centered Care – Transitions in Care
	Population and Community Health
	Physician Role in Health Care Systems
**Practice Based Learning and Improvement (PBLI)**	
	Evidence Based and Informed Practice
	Reflective Practice and Commitment to Personal Growth
**Professionalism (PROF)**	
	Professional Behavior
	Ethical Principles
	Accountability/Conscientiousness
	Well-Being
**Interpersonal and Communication Skills (ICS)**	
	Patient and Family Centered Communication
	Interprofessional and Team Communication
	Communication within Health Care Systems

^*^Competencies are new to Milestones 2.0 and are pediatric nephrology specific

**Fig. 1:**
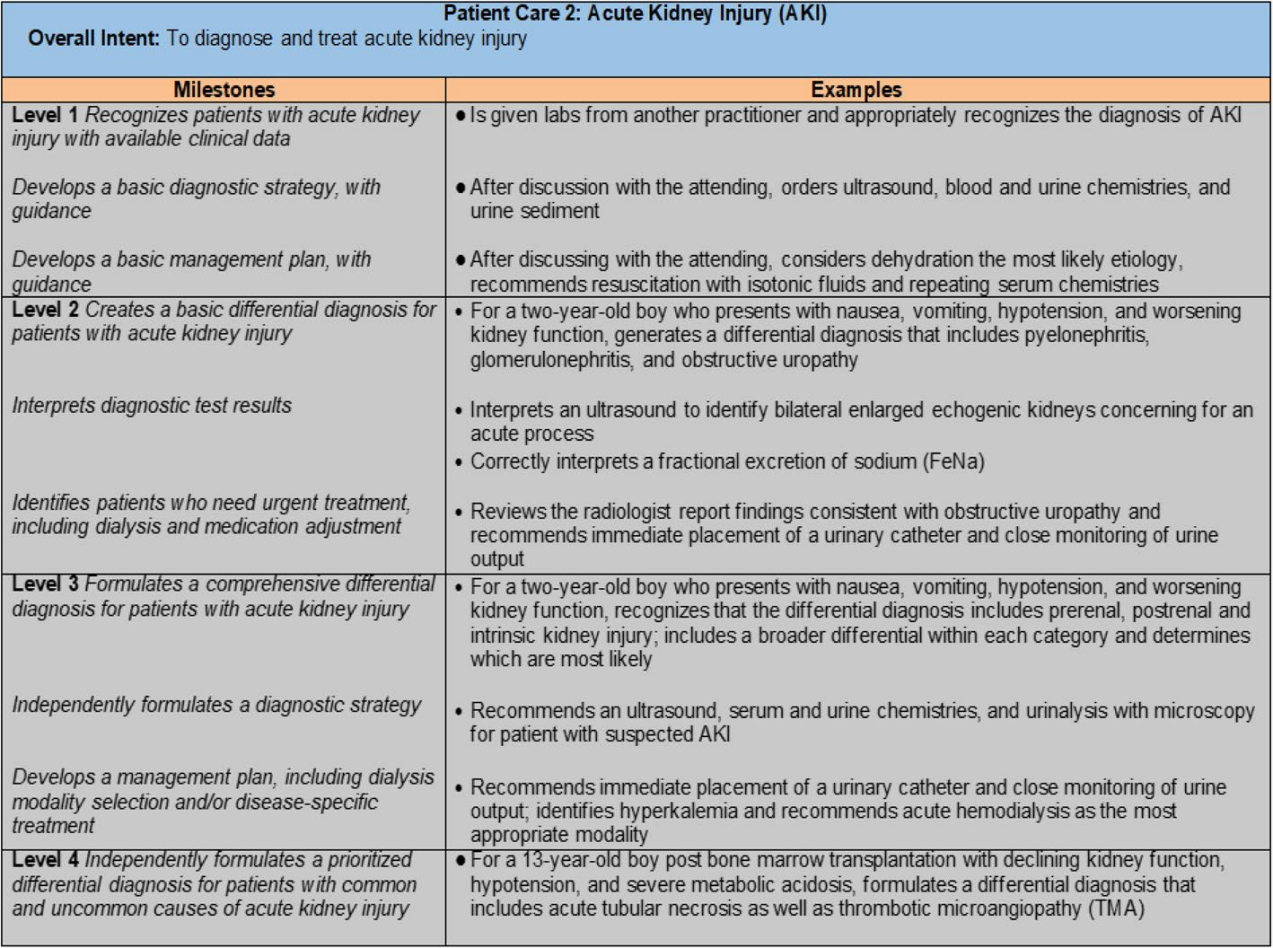
Example from supplemental guide providing context for subcompetency Patient Care 2 (PC2). Used with permission from the Accreditation for Graduate Medical Education (ACGME)

The Supplemental Guide was then revised to match the new Milestones and to provide concrete and realistic examples of how a trainee might demonstrate mastery of a particular skill (Fig. 1). This work occurred both in-person and through virtual meetings to determine consensus agreement. All additions or changes to competencies and subcompetencies were discussed with the full work-group present, in person. Supplemental guide materials were updated and created in smaller subgroups with the final proposals shared with the larger group for additional suggestions and final approval. The overall goal of these revisions was to make the Milestones clearer and more user-friendly, such that all members of the educational team would interpret and implement them similarly. This, in turn, would enhance consistency in the evaluation process and minimize inter-individual variability in assessment.

Community feedback was then solicited through public review and dissemination of the proposed Milestones 2.0 through a national pediatric nephrology training program director subcommittee. They were also reviewed at the Pediatric Academic Societies meeting in Washington D.C. in May of 2023. After the open commentary period, final revisions were made, and Milestones 2.0 were officially implemented on July 1, 2023.

## Results

A list of the final pediatric nephrology subcompetencies can be reviewed in Table 1. The complete pediatric nephrology Milestones 2.0 can be found on the ACGME website with links to the finalized supplemental guides [3].

## Discussion

### Expected improvements with implementation of milestones 2.0

The goals of Pediatric Nephrology Milestones 2.0 include creating a shared mental model and transparency in expectations for performance, growth in learning and fair assessment. Increased ease of feedback delivery and more granular assessments will ideally lead to improvements in the quantity and quality of feedback received by fellows. Milestones 2.0 has been designed to promote a growth focused mindset, more equitable evaluation, and the ability to provide both just-in-time and summative feedback.

### Summary of major changes from milestones 1.0 to 2.0

One significant difference between Milestones 1.0 and Pediatric Nephrology Subspecialty Milestones 2.0 is the addition of specific nephrology topic-based subcompetencies within the Clinical Skills (CS) core competency (Table 1). In addition to “organization and prioritization of patient care” and “competence in procedures” subcompetencies, seven key nephrology topics were added within the patient care core competency category. The goal of these additions is to improve the Milestones ability to more accurately reflect how fellows can acquire skills within different realms of nephrology at different times. For example, a fellow may be functioning quite independently in caring for patients with hypertension but still need more support and guidance in the care of patients who have received a transplant. Early recognition of slower than expected development along the Milestone’s growth trajectory in a specific clinical topic would ideally permit adjustments to a fellow’s clinical exposure and teaching regarding that particular learning area.

**Fig. 2:**
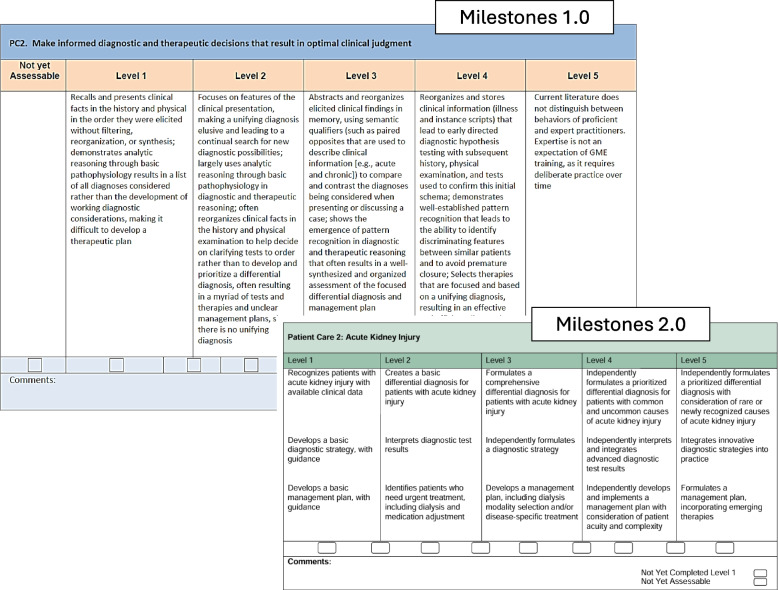
Comparison of Patient Care 2 subcompetency between Milestones 1.0 & 2.0 highlighting simplification of wording and length in Milestones 2.0. Used with permission from the Accreditation for Graduate Medical Education (ACGME)

Simplification of jargon and decrease in the length of milestones descriptions was another goal of the revision. The pediatric nephrology working group aimed to change milestones to be quickly understandable with the option for evaluators to refer to the supplemental guide for further clarification and “real-life” examples of a particular milestone (Fig. 2).

#### Growth focused language

The wording and tone of Milestones and related materials was revised to reflect a more growth-focused mindset to highlight the acquisition of new skills rather than using negative language to describe earlier skill levels. As every level of the Milestones is an expected steppingstone towards acquisition of skills needed for independent practice, trainees assessed as Level 1 or 2 should not interpret this as failure, rather as having a basic skillset that will serve as a foundation from which to build more complex skillsets going forward during their training.

For example, the PC-2 Level 2 Milestones 1.0 description read:

*“often reorganizes clinical facts in the history and physical examination to decide on clarifying tests to order rather than to develop and prioritize a differential diagnosis, often resulting in a myriad of tests and therapies and unclear management plans.”*

PC-2 Level 2 within Milestones 2.0 now states

*“… creates a basic differential diagnosis, interprets diagnostic test results, identifies patients who need urgent treatment*…”

and Level 3 reads:

“…*formulates a comprehensive differential diagnosis, independently formulates a diagnostic strategy, develops management plan with disease specific treatment*…”

Though reflecting the same skillsets in both versions, Milestones 2.0 now highlights how a fellow progresses from *“basic” differential diagnoses to “comprehensive” differential diagnoses and from simply “interpreting test results”* to now *“formulates diagnostic strategy and develops a management plan.”* As a learner progresses from Level 2 to Level 3, they will naturally be moving away from *“myriads of tests and unclear management plans”* described in Milestones 1.0 but in the revised version, focus is on acquisition of skills that will lead to superior proficiency rather than highlighting the negative impacts of their earlier skill level.

#### More equitable evaluation

Currently, only a subset of faculty may serve on a given fellow’s clinical competency committee (CCC). Often, CCC members have not personally witnessed the fellow demonstrate acquired skills in every area. Therefore, CCC committees rely on submitted feedback from faculty working directly with fellows. Oftentimes some faculty provide more feedback than others which can lead to a skewed perception of a fellow’s skill through the lens of only a few evaluators. Though best intentioned, this system is quite vulnerable to inferior quality and inequitable assessment.

If there is not enough quantity of specific feedback provided, the CCC may resort to “guestimating” the fellow’s degree of achievement within some milestones. This practice also involves significant risk of misrepresentation of the fellow’s actual achievements secondary to bias or misinterpretation of limited feedback.

The granularity and simplicity of Milestones 2.0 should enable evaluators to provide specific feedback more quickly to fellows and create more data-driven global assessments by program leadership. If feedback is less time and energy-consuming, we expect increased participation from evaluators and therefore increased data by which to create accurate summative feedback for fellows. This has been demonstrated by improvement in satisfaction of frequency, timeliness and quality of feedback by a surgical subspecialty program who created a similar simplified feedback program [9].

#### Opportunities for use for just in time feedback

**Fig. 3:**
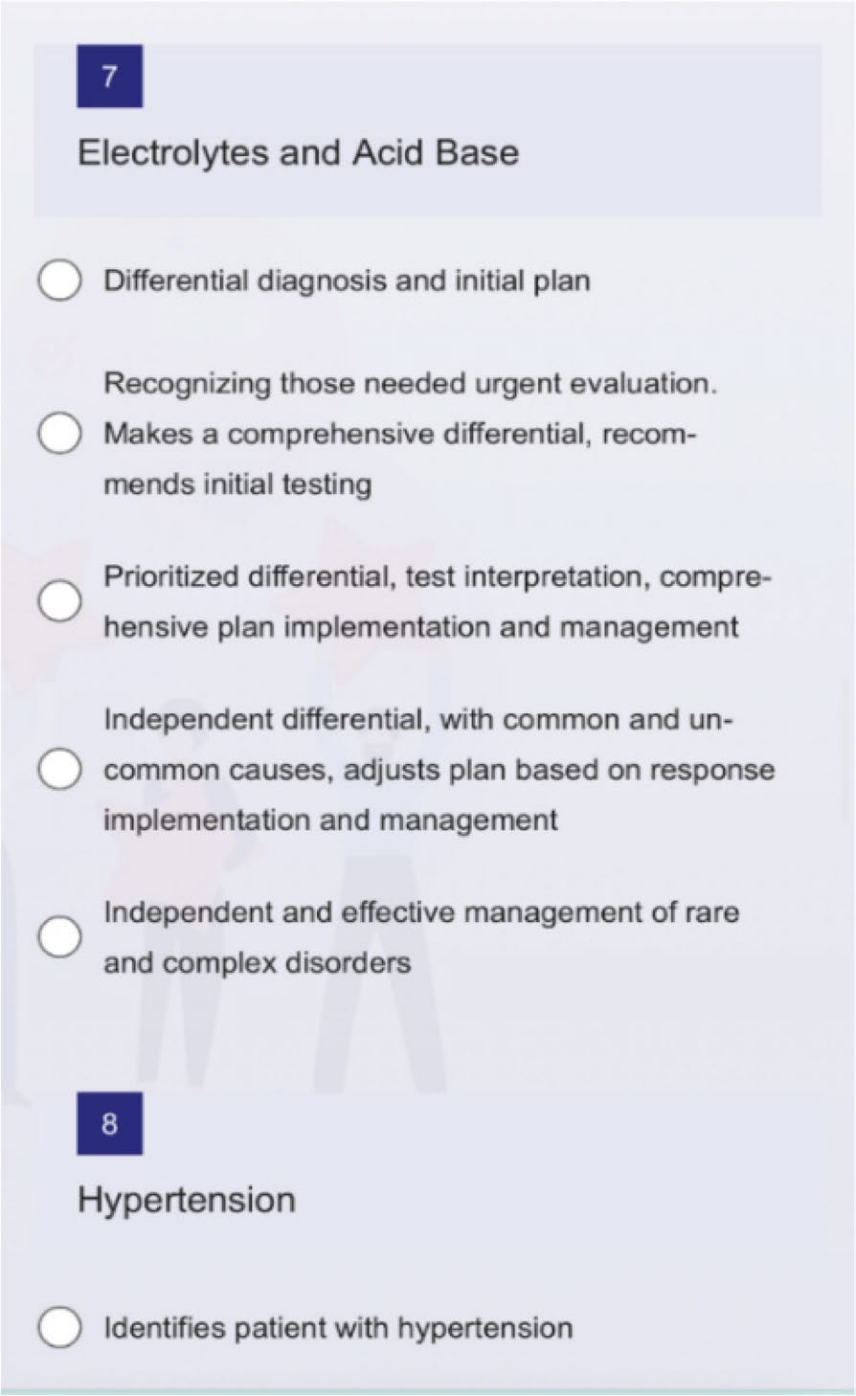
Example feedback survey for patient care core competency, acid base subcompetency on an evaluators phone screen

One author’s institution is piloting a program using a Milestones-based system for just-in-time feedback. They created an electronic evaluation form with a generic survey generation tool including each subcompetency within the PC and MK core competencies as one of eleven potential areas for evaluation (Fig. 3). The survey offers evaluators five assessment options which correlate to the respective Milestones within the specific subcompetency. After a clinical encounter, a fellow can show their supervisor a QR code on their hospital identification badge or provide a link to the electronic survey. Using a mobile device, the QR code can be quickly scanned or the electronic survey link quickly accessed to facilitate a timely evaluation for the discrete clinical encounter.

**Fig. 4:**
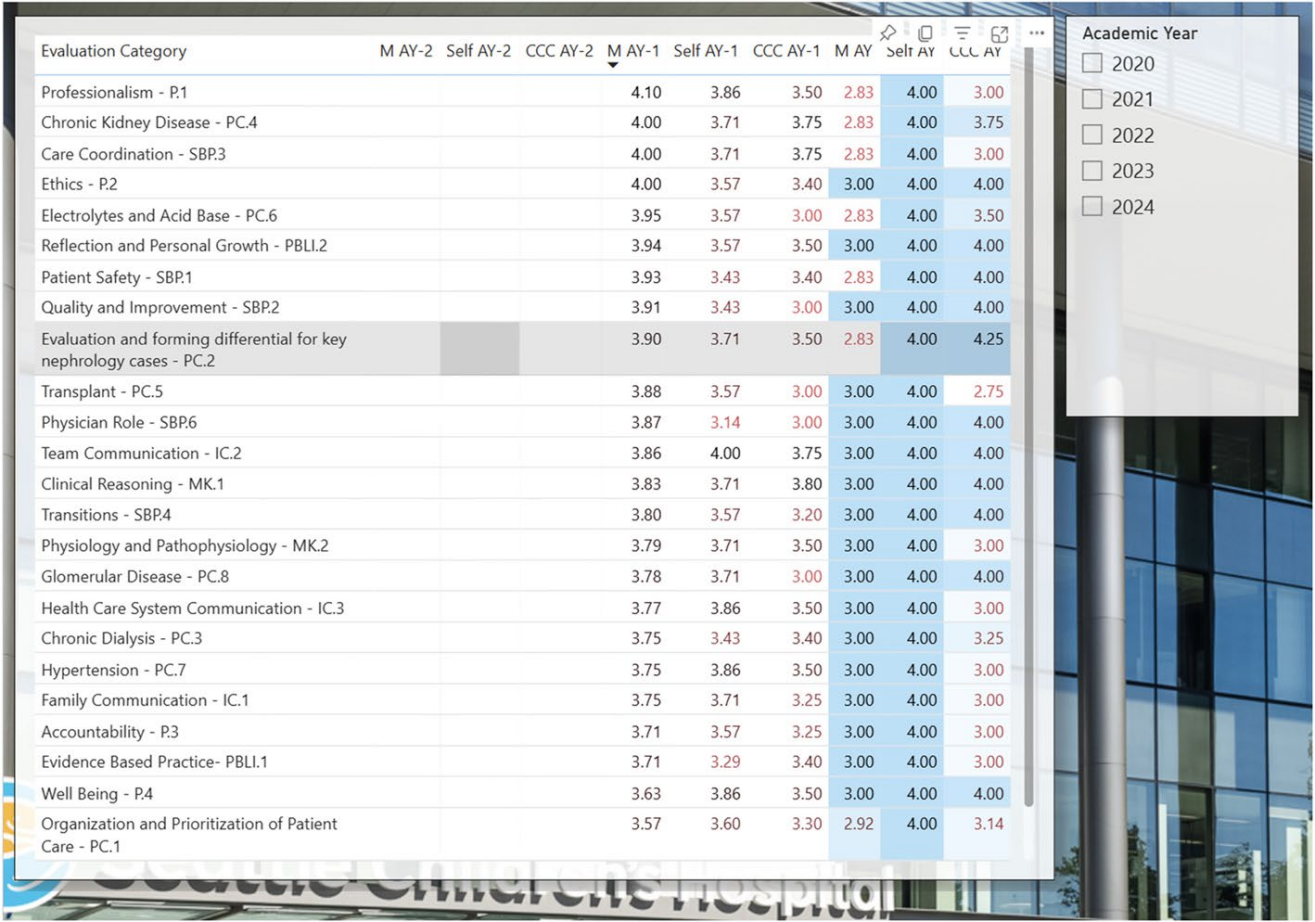
Collated subcompetency scores for an individual fellow based on faculty evaluations and self-assessment. *Please note these scores are fabricated for demonstration purposes only and do not reflect the performance of any actual trainee

For example, if an attending and fellow cared for a patient with electrolyte abnormalities that day, the attending would fill out the portion of the survey under the “Electrolytes and Acid Base” option. The evaluator can simply provide a numeric score that corelates best with the fellows achieved Milestone and also has the option to leave written feedback if desired. Ideally, every fellow receives just in time feedback at least once a day which in turn provides a significant amount of data to curate and review at regular intervals. Fellows and program directors can use the results of these electronic evaluations to identify strengths, areas of progress, and content domains where additional growth needs to be supported. Feedback is provided anonymously and can be viewed with summarizing visual graphics after curation of many evaluations, including self-evaluation by fellows done at regular intervals (Fig. 4). At the author’s institution, in order provide easy access to evaluation tools and frequent reminders to provide feedback, QR codes linking to the survey are posted in clinic (on the doorframe near the exit and on computer monitors) in addition to being on the trainee’s badge and available online.

#### Opportunities for self-evaluation

An evaluation system like that described above can also be implemented for fellow self-evaluation permitting comparison between perceived skill level and compiled evaluation results from faculty. At one author’s institution this is implemented as a biannual practice at this program to highlight discrepancies between perceived and actual capabilities. Many trainees suffer from imposter syndrome and underestimate their capacity. This practice may help build confidence in their ability to reach independence [10, 11].

#### Opportunities for programmatic assessment and improvement

**Fig. 5:**
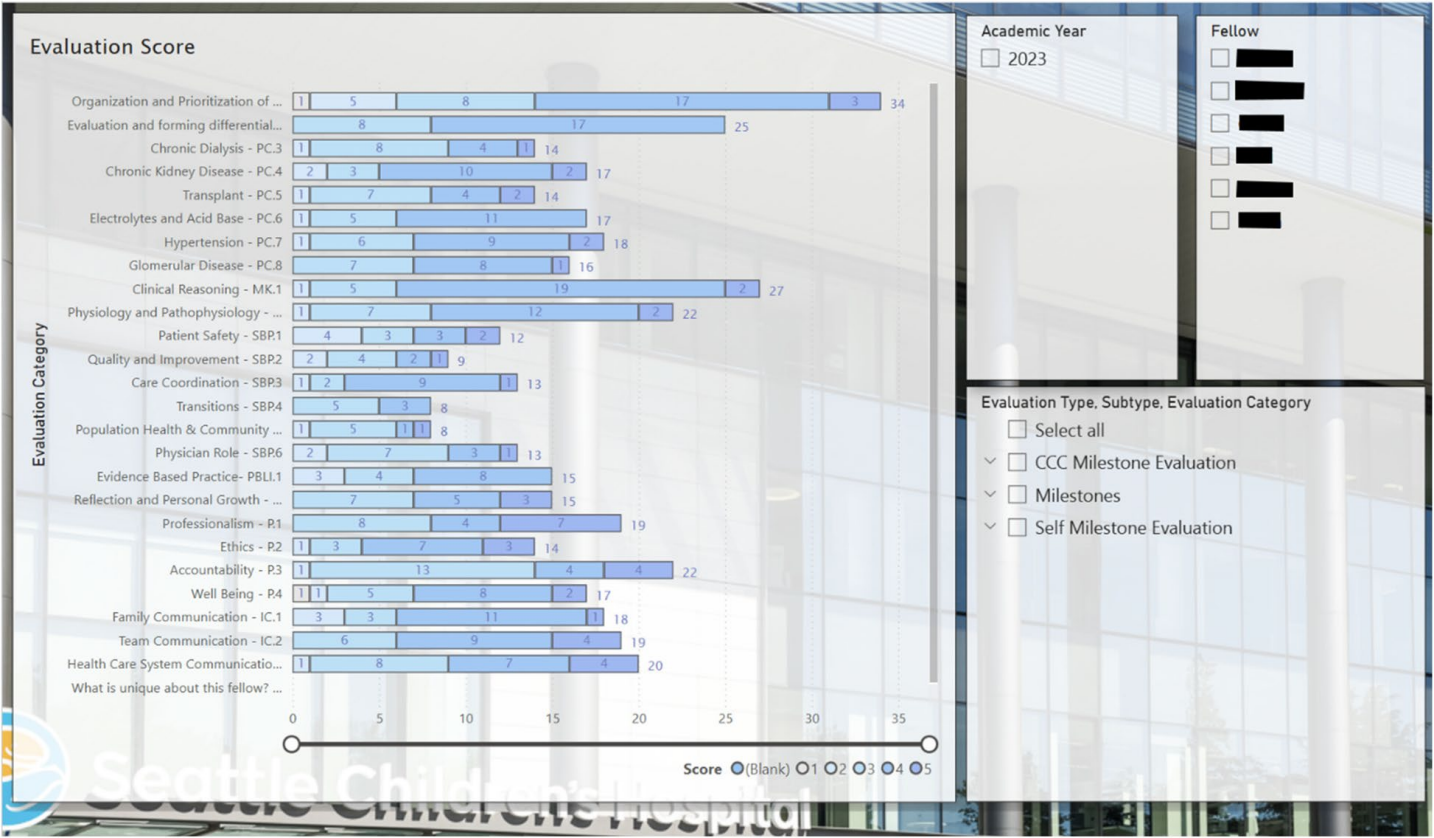
Collated data assessing the number of evaluations obtained amongst all fellows in a single program to be used for assessment of programmatic strengths and need

The cumulative Milestones data can also identify areas where there is an overall lack of exposure in certain fields of nephrology at a programmatic level (Fig. 5). For example, if one or many trainees have only minimal evaluations for the patient care glomerulonephritis subcompetency, efforts can be made to increase experience and exposure to patients with this condition and supplement with educational curriculum.

#### Use of the milestones for performance improvement plan

**Fig. 6:**
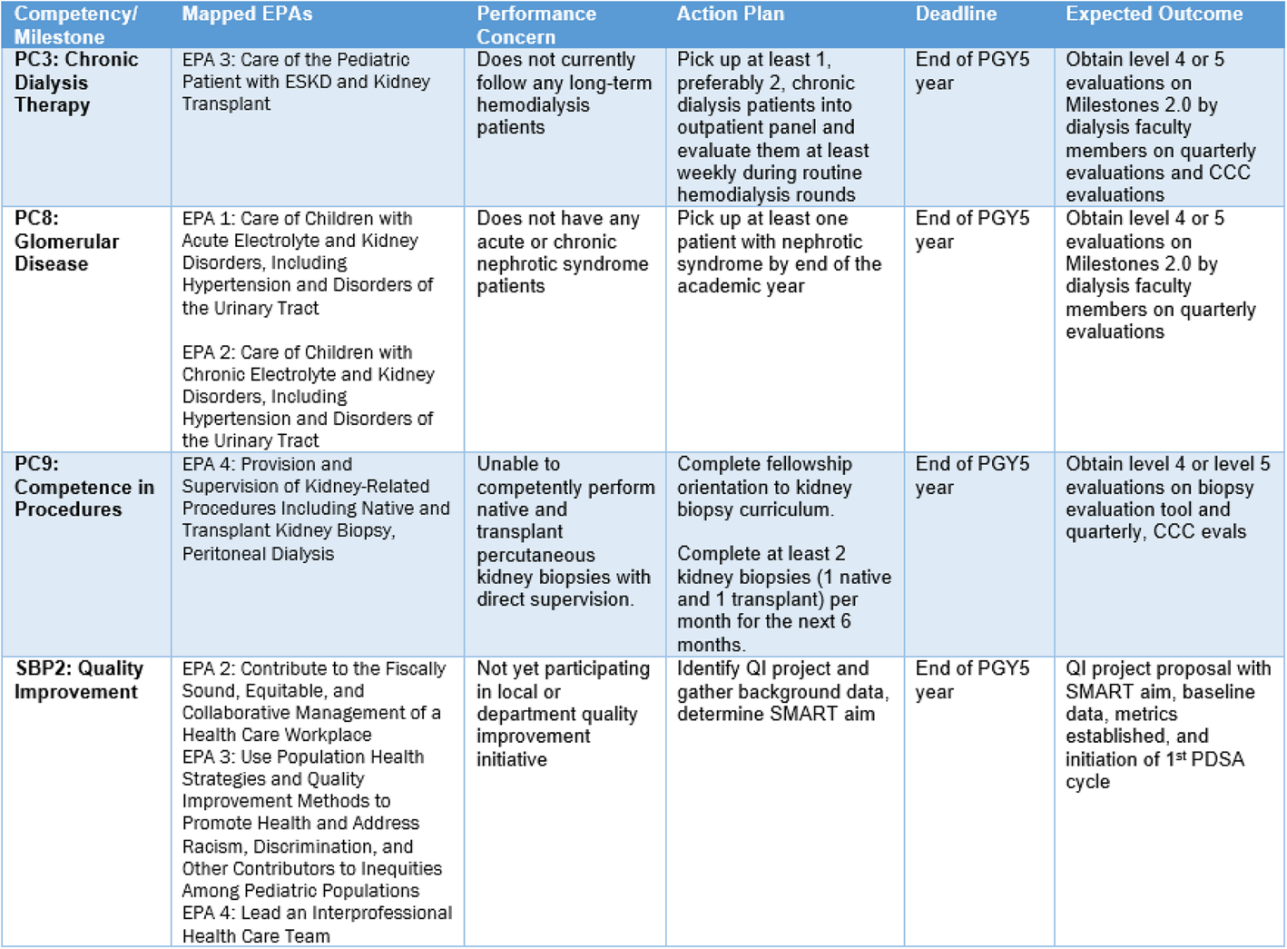
Performance Improvement Plan (PIP) generated from Milestones based assessments. *This PIP was created for demonstration purposes only and does not reflect PIP for any actual trainee

A different pediatric nephrology fellowship program used the Milestones 2.0 to create a performance improvement plan (PIP) for fellows in need of additional support or remediation (Fig. 6). Consistency in expectations for skill acquisition and verbiage used to describe them will ideally create improved understanding by fellows regarding where they stand in current skill acquisition. The PIP also highlights specific performance concerns with discrete action plans for each competency with time-bound expectations.

## Conclusions

Pediatric Nephrology Milestones 2.0 was developed via the joint efforts of the ACGME, invested pediatric nephrologists/educators, and current pediatric nephrology fellows to transform Milestones 1.0 into a more effective tool. This manuscript provides a window into the development of Milestones 2.0 for pediatric nephrology and concrete examples of how it might be implemented in routine practice. We expect that every program will adapt these tools to meet individual program needs.

The next logical steps include gathering data regarding efficacy and satisfaction of Milestones 2.0. Quantitative measures of the amount of feedback provided to trainees would be useful as would qualitative data regarding faculty and trainee perceived efficacy and utility of feedback. Ideally, with the improved standardized nature and clearer descriptions of trainee expectations at each Milestone level, Milestones 2.0 will reduce bias in fellow assessment and lead to a more equitable educational experience for all trainees. Further evaluation of the Milestones 2.0 impact on bias in graduate medical education should be prioritized.

## Data Availability

Materials regarding the Milestones are publicly available at https://www.acgme.org/milestones/resources/.

## Abbreviations

ACGME: Accreditation Council for Graduate Medical Education
CBME: Competency based medical education
ABMS: American Board of Medical Specialties
PC: Procedural skills
MK: Medical knowledge
SBP: Systems-based practice
PBLI: Practice-based learning and improvement
PROF: Professionalism
ICS: Interpersonal communication skills
APPD: Association of Pediatric Program Directs
ASPN: American Society for Peidatric Nephrology
COPS: Council of Pediatric Subspecialties
ABP: American Board of Pediatrics
CCC: Clinical Competency Committee
PIP: Performance Improvement Plan
